# Imperforate Hymen, a Case

**Published:** 1887-07

**Authors:** C. O. Weller

**Affiliations:** Austin, Texas


DANIEL'S

### Texas Medical Journal.

Published Monthly.—Subscription $2.00 a Year.

Vol. 3. AUSTIN, JULY, 1887. No. 1.

### Original Articles.

CONTRIBUTED EXCLUSIVELY TO THIS JOURNAL.

*The Articles in this Department are accepted and published with the understanding that we are not responsible for, nor do we indorse the views and opinions of the writers by so doing.*

### IMPERFORATE HYMEN.

*By C. O. Weller, M. D. Avstin, Texas.*

For Daniel's Texas Medical Journal.

In the night of March 16, 1887, I was called to see ———, a girl thirteen years of age, living five miles in the country. I found my patient a stout, well developed and healthy looking girl. Her mother informed me that since August, 1886, she had had monthly symptoms,as if the menstrual function were about to be established, yet there had never been any catamenial flow. After that condition of things had existed for several months, the periodical suffering had increased to such a degree that the family physician was consulted. He prescribed remedies designed for the establishment of the menstrual discharge, but without the result desired. The night that I was called, the girl had been in great pain, suffering from frequent bearing down efforts, and also difficult and frequent micturition. The pain and nervous disturbance were very great; so much so, that quite a severe convulsion was the result.

The family physician was now dispatched for in haste, but being unwell, he referred the messenger to me. Upon arrrival I found the patient resting comparatively easy from the effect of a dose of morphine, left on a previous occasion by her physician, and at this time administered by her mother. In addition to the foregoing his tory, I was also informed that a gradual enlargement of the patients abdomen had been noticed during the past two or three months. I explained the necessity of a thorough examination, which was consented to. Upon palpation of the abdomen, I found a hard resisti g body, dull on percussion, extending from the symphysis pubis to the umbilicus, and much the shape of a uterus occupied by a five or six months fœtus.

There having been considerable difficulty in voiding the urine, I did not know what part a distended bladder might play in this condition of things. I consequently introduced a catheter as a means of diagnosis, and drew off a few ounces of urine, without however any perceptible effect on the size of the hypogastrium. Passing my finger to the ostium vaginæ, I found the opening completely closed by a dense, resisting and bulging membrane.

Taking the history of the case in connection with the physical conditions present, my diagnosis was readily made, and communicated to the girls mother, namely, retained menses from an imperforate hymen. It now being late, I took my leave, after having informed the parents, that I would call the next morning with their regular medical attendant, and should he agree with me as to the nature of the case, we would institute means for the relief of their daughter.

The next day Dr. Graves and myself visited the patient together. The doctor concurred with me in my diagnosis, and proposed a mode of relief. The uterus having attained its present state by a gradual filling and distention, during the past eight months, it was thought best not to evacuate its contents too rapidly, but make a small puncture and withdraw the fluid slowly, giving it time to contract and adapt itself to its changed condition. This was accordingly done, and after about one quart of black, bloody fluid passed, the opening was closed by the introduction of a small carbolized tent. Returning the next day the mother informed us, the tent came away during the night, when the girl was evacuating her bladder, and that a large quantity, (two chambers full, she said) of the bloody fluid passed from her. The first opening was now enlarged, and the hymen incised through its whole extent, and the vagina and womb thoroughly washed out with a solution of carbolic acid in warm water. There was no offensive odor about the discharge. A piece of absorbent cotton well rolled in iodoform was placed between the edges of the incision. At our visit the next day we found some fetor connected with the vaginal flow, which still continued black. The parts were now well irrigated with warm carbolized water, and the washing continued until all offensive smell had ceased, and the water returned clear. Her mother was now instructed in the use of the syringe, and directed to use warm carbolized water, freely, three time in the twenty four hours. This she did, and the girl has made a good recovery, with only slight fever during two days following the evacuation of the retained menses. Three months now have elapsed; she is well, and her menstrual function is being performed normally.

